# Evaluation of Root Films with *Bacillus subtilis* for Establishment and Growth Promotion in Tomato

**DOI:** 10.3390/plants14243716

**Published:** 2025-12-05

**Authors:** Guadalupe Oyoque-Salcedo, Oscar Giovanni Gutiérrez-Cárdenas, Omar Fabián Hernández-Zepeda, Juan Carlos Raya-Pérez, Jorge Covarrubias-Prieto, Glenda Margarita Gutiérrez-Benicio, María Valentina Angoa-Pérez, Ernesto Oregel-Zamudio, César Leobardo Aguirre-Mancilla

**Affiliations:** 1 Instituto Politécnico Nacional, Centro Interdisciplinario de Investigación para el Desarrollo Integral Regional (CIIDIR), Unidad Michoacán, Justo Sierra 28, Col. Centro, Jiquilpan 59510, Michoacán, Mexico; goyoque@ipn.mx (G.O.-S.); vangoa@ipn.mx (M.V.A.-P.); 2 Tecnológico Nacional de México/Instituto Tecnológico de Roque, Carretera Celaya–Juventino Rosas km 8, Celaya 38110, Guanajuato, Mexico; juan.rp2@roque.tecnm.mx (J.C.R.-P.); jorge.cp@roque.tecnm.mx (J.C.-P.); glenda.gb@roque.tecnm.mx (G.M.G.-B.); 3 Genómica Alimentaria, Universidad de La Ciénega del Estado de Michoacán de Ocampo, Sahuayo 59103, Michoacán, Mexico; oggutierrez@ucemich.edu.mx (O.G.G.-C.); ofhernandez@ucemich.edu.mx (O.F.H.-Z.)

**Keywords:** bioinoculant, *B. subtilis*, PGPR, root colonization, *Solanum lycopersicum*

## Abstract

The presence of *Bacillus subtilis* on tomato roots contributes to plant growth promotion, which depends on its ability to establish in the roots. Edible-film formulations have emerged as effective carriers for beneficial bacteria. In this study, we evaluated film formulations based on guar gum, glycerol, and candelilla wax incorporating *B. subtilis* for root application in tomato seedlings to stimulate plant development. Sixteen film formulations were prepared and applied to seedling by dipping root; plants were grown under a 16/8 h photoperiod. At 60 days, growth parameters (plant height, leaf number, chlorophyll content, fresh and dry weights) were measured, along with *B. subtilis* on roots, and the soil degradation of the selected film. Treatments were: seedlings with *B. subtilis* at two doses (10^6,12^ CFU/mL (B6, B12), film without bacteria (P), films with *B. subtilis* (P1–P16), and untreated (TST). Among the films, formulation 9 (guar gum 0.6%, candelilla wax 0.15%, glycerol 0.15% and *B. subtilis* 20%, 1 × 10^12^) significantly increased shoot and root biomass and supported higher root colonization of *B. subtilis* (2.3 × 10^7^ CFU/g). The film degraded in soil within 15 days, while the inoculant maintained high viability (6.3 × 10^8^ CFU/mL) after 8 months at 4 °C. These results highlight film formulation 9 as a promising bioinoculant for tomato cultivation.

## 1. Introduction

Plant-growth-promoting rhizobacteria (PGPR) are key components of biofertilizers due to their ability to enhance soil health and crop productivity. In tomato (*Solanum lycopersicum*), inoculation with strains such as *Bacillus subtilis* promotes phytohormone synthesis, nutrient solubilization, and overall yield improvement [1,2]. Their use also reduces reliance on chemical fertilizers, contributing to more sustainable production systems.

*Bacillus subtilis* (Ehrenberg 1835) Cohn 1872 is one of the most studied microbial inoculants as a pathogen control agent, plant growth promoter, and soil improver [3,4,5]. For example, the *B. subtilis* strain Pn1 inhibited *Fusarium solani* growth by 72.08% in vitro. In *Panax notoginseng* (Burkill) coinoculated (10^8^ CFU/mL and 1 × 10^7^ spores/mL), it reduced disease caused by *F. solani* to 35% compared with 85% in plants without the bacterium, and it stimulated plant development under field conditions. In roots, Pn1 activated biosynthetic pathways of phytohormones (cytokinins, auxins, gibberellins), phenylpropanoids, and flavonoids, contributing both to protection and growth promotion [6].

The bacterium is commonly applied to soil or roots, individually or in combination with other strains, to improve plant protection. For instance, individual or mixed (1:1 *v*/*v*) applications of *B. subtilis* PTB185 and *B. pumilus* PTB180 (1 × 10^7^ CFU/mL) were sprayed (15 mL per plant, repeated after 2 days) on 4-week-old tomato (*Solanum lycopersicum* L.) plants. Plants were later inoculated with *Botrytis cinerea* Pers., 1801 (20 mL, 1 × 10^6^ conidia/mL). *B. subtilis* PTB185 reduced disease incidence by ≈52% and severity by ∼20%, while the mixture showed reductions of ∼55% and ∼25%, respectively, compared with the control (∼83% and ∼42%). PTB185 produced fengycins, surfactins, and iturins [7].

Adjuvants in formulations have also been implemented to favor bacterial retention at the desired site. [1] formulated *B. subtilis* (9 × 10^8^ CFU/mL) with carboxymethylcellulose and talc as adherent. Tomato seeds (var. S22) were soaked for 24 h in water with talc (4 g/kg seed). At 30 days, seedlings were transplanted, and the product was applied to soil (2.5 kg/ha), with subsequent sprays (0.1%) at 30, 60, and 90 days after transplanting. This treatment reduced fungal diseases caused by *Fusarium oxysporum* (Schltdl., 1824), *Pythium aphanidermatum* (Edson) (Fitzp., 1923), *Colletotrichum capsici* (Syd.) (E.J. Butler & Bisby 1931), and *Sclerotium rolfsii* (Sacc., 1911) and maintained a yield of 11.73 t/ha, equivalent to chemical control (11.8 t/ha).

Among recent strategies, Wang et al., 2025 [8] evaluated chitosan microspheres loaded with *B. subtilis* ACCC1089 to promote growth of *Lactuca sativa* L. var. ramosa and shoot and root biomass increased by 88% and 47%, respectively. In the rhizosphere, fractions of Firmicutes (0.7%), Bacteroidetes (0.7%), and Proteobacteria (0.8%) were identified.

Similarly, Saberi et al., 2020 [9] evaluated *B. subtilis* Vru1 (1 × 10^10^ CFU g^−1^) nanoencapsulated in sodium alginate (1.5%), bentonite (4%), starch (3%), and TiO_2_ (10 ppm), applying 20 mL/kg of soil in pots under greenhouse conditions. The nanoencapsulated population showed a lower initial release (~5 log CFU g^−1^) versus the free bacterium (~9.5 log CFU g^−1^); however, it reached ~9 log CFU g^−1^ on roots by 45 days, whereas the free bacterium declined to ~4 log CFU g^−1^. Plants treated with the nanoencapsulated formulation had higher fresh/dry weights of shoots and roots, as well as greater height, even under challenge with *Rhizoctonia solani* (J.G. Kühn, 1858).

Despite advances in different application modes for *B. subtilis*, long-term toxicity of formulations and their interaction with soil components still need evaluation so that efficacy and availability are not compromised. Seed coating is considered a method that reduces inoculant use and significantly increases shoot biomass, favors early contact of bacteria with roots, and enables establishment [10,11]. However, some studies note limitations. [12], for example, evaluated the effect of applying *Pantoea* spp. (ITSI10, BTRH79) and *Pseudomonas* sp. (MIXRI75) individually or combined, either on seeds or soil, to promote growth of Italian ryegrass (*Lolium multiflorum* var. Taurus). The combination significantly increased shoot and root biomass, correlating with higher bacterial density in the rhizosphere, roots, and shoots, compared with seed imbibition, which showed lower biomass, colonization, and degradation.

Commercial and research products also face limitations: short shelf life, low efficacy under variable cultivation conditions, limited competitiveness against native microbiota, and functionality dependent on farmer expertise [13,14,15]. Plant growth promoting rhizobacteria (PGPR) efficacy depends largely on their ability to colonize the rhizosphere or establish on roots at densities sufficient to promote growth [16,17] though resident microbiota and abiotic factors challenge this process [18,19,20].

Therefore, formulations should ensure inoculant viability long enough to establish in the rhizosphere and exert beneficial effects [21,22]. In this context, films incorporating microorganisms have been developed for phytopathogen control or to increase survival. Examples include an *Aloe vera* (100%) film with glycerol (10 g/L) and glucose (0.1 M) containing *Lactobacillus paracasei* (Coollin et al. 1989) (10.4 log CFU g^−1^) that maintained viability (10.9 log CFU g^−1^) and controlled *Colletotrichum gloeosporioides* in vitro (Penz.) (Penz. & Sacc., 1884) in vitro [23]; a sodium alginate (2% *w*/*v*) and glycerol (0.24 g/g alginate) biofilm with *Wickerhamomyces anomalus* (E.C. Hansen, 1889) and *Pichia membranifaciens* (E.C. Hansen, 1904) (≈6 log CFU/cm^2^) that maintained 75% and 60% viability, respectively, and controlled *B. cinerea* and *Penicillium italicum* (Wehmer, 1894) on apples [24] and a carboxymethylcellulose (1% *w*/*v*) and glycerol (50% *w*/*w*) film with *Lactobacillus acidophilus* (Johnson et al. 1980), *L. casei* (Orla-Jensen 1916), *L. rhamnosus* (Hansen 1968), and *Bifidobacterium bifidum* (Tissier 1900) (10^9^ CFU/g) that maintained ≈10^7^ CFU g^−1^ at 4 °C for 42 days, with greater loss at 25 °C [25].

The capacity of *B. subtilis* to control fungi on tomato roots and other crops is widely recognized [26], in some cases with efficacy similar to chemical fungicides—as with *B. subtilis* BI600 controlling *F. oxysporum f.* sp. *radicis cucumerinum* in cucumber (80%), comparable to chemical control (90%) [27]. Its role as a growth promoter in tomato has also been documented [28]. The strain *B. subtilis* PTS-394 colonized tomato roots 7 days after inoculation, increasing plant height by 8.9% and root weight by 18.3% relative to control. This effect on the rhizosphere microbiota was transient; by 14 days no significant differences with control were recorded [29].

Despite advances, the challenge remains to identify carriers that maintain inoculant viability, facilitate host colonization, and preserve activity throughout the crop cycle. One strategy is encapsulation in sodium alginate crosslinked with CaCl_2_, which maintained high bacterial viability (5.3 × 10^8^ CFU/mL) for five months in the lyophilized product. In lettuce seedlings, it promoted increases in root (~76%) and shoot (~64%) length and favored rhizoplane colonization (10^7^ CFU/cm) versus free cells (2 × 10^3^ CFU/cm), with gradual bacterial release [30].

Although laboratory adaptive evolution can yield *B. subtilis* strains with higher PGPR and biocontrol potential, the real test is field validation, where variable rhizosphere conditions determine success [31]. Moreover, the delivery method conditions establishment: in sorghum, seed coating favored Gram-positive bacteria, whereas root soaking or drench worked better for Gram-negative bacteria, indicating the need to match application method to the inoculant to optimize colonization and performance [32].

Under gnotobiotic conditions, soaking tomato seedlings for 10 min in a *B. subtilis* suspension (1 × 10^6^ CFU/mL) enabled pre-colonization of root tips at early stages. The bacterial population in the matrix reached ~1 × 10^9^ CFU/g at 15 days, and sporulation was rapid (~55% on day 2 and ~88% on day 15), suggesting inoculant persistence albeit with lower metabolic activity at later stages [33] These findings support that early application of inoculants facilitates establishment and persistence in the rhizosphere.

Therefore, it is necessary to keep exploring strategies that ensure direct and immediate contact of bacteria with roots, favoring rapid, stable, and functional colonization throughout crop development. In this context, films formulated with microorganisms are an effective alternative for root inoculant application. In previous studies, a film composed of guar gum, candelilla wax, and glycerol with *B. subtilis* controlled *Rhizopus stolonifer* on strawberries and extended shelf life [34]. Building on these results, and considering the innocuity of the formulation’s ingredients, new films were designed as vehicles to favor *B. subtilis* establishment and promote growth of tomato plants.

## 2. Results

### 2.1. Evaluation of Root Films for the Promotion of Tomato Growth

**Figure 1:**
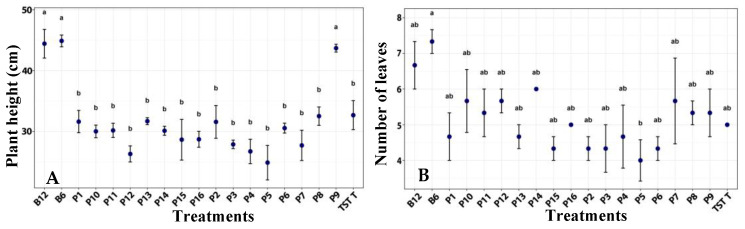
Height (**A**) and leaves of number (**B**) of tomato plants under a photoperiod, after the application of treatments. B12 (*B. subtilis* 1 × 10^12^ CFU/mL), B6 (*B. subtilis* 1 × 10^6^ CFU/mL), TST T (untreated plants), P1–P16 (film formulation 1–16). Values are mean ± SE (n = 10). Different letters indicate significant difference among treatments (Tukey’s test, *p* ≤ 0.05).

Significant differences were observed in plant height. Plants treated with film 9, as well as those treated with both *B. subtilis* concentrations (B12, B6), showed greater height compared with the other treatments. Leaf number did not differ significantly among treatments (Figure 1).

**Figure 2:**
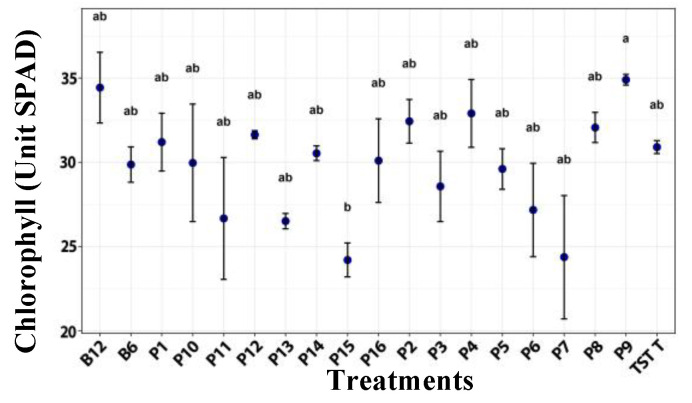
Chlorophyll in tomato plants under a photoperiod, after the application of treatments. B12 (*B. subtilis* 1 × 10^12^ CFU/mL), B6 (*B. subtilis* 1 × 10^6^ CFU/mL), TST T (untreated plants), P1–P16 (film formulation 1–16). Values are mean ± SE (n = 10). Different letters indicate significant difference among treatments (Tukey’s test, *p* ≤ 0.05).

For chlorophyll, no significant statistical differences were detected among treatments; however, plants treated with film 9 tended to show higher chlorophyll content (Figure 2).

**Figure 3:**
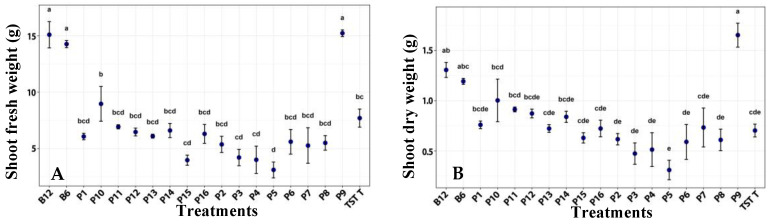
Shoot fresh weight (**A**) and dry weight (**B**) of tomato plants under a photoperiod, after the application of treatments. B12 (*B. subtilis* 1 × 10^12^ CFU/mL), B6 (*B.* subtilis 1 × 10^6^ CFU/mL), TST T (untreated plants), P1–P16 (film formulation 1–16). Values are mean ± SE (n = 10). Different letters indicate significant difference among treatments (Tukey’s test, *p* ≤ 0.05).

Shoot fresh weight in plants treated with film 9 and with both bacterial suspensions (B6, B12) was significantly higher than in the other treatments. Plants treated with film 9 also showed higher shoot dry weight than plants receiving the other films and the control (Figure 3).

**Figure 4:**
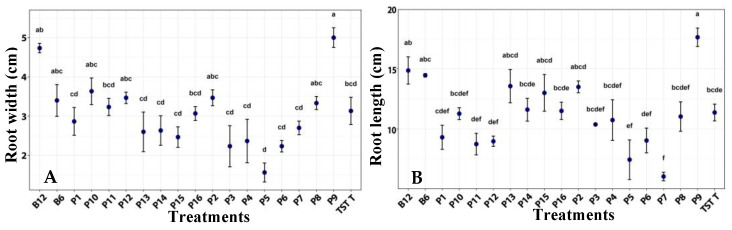
Root width (**A**) and length (**B**) of tomato plants under a photoperiod, after the application of treatments. B12 (*B. subtilis* 1 × 10^12^ CFU/mL), B6 (*B.* subtilis 1 × 10^6^ CFU/mL), TST T (untreated plants), P1–P16 (film formulation 1–16). Values are mean ± SE (n = 10). Different letters indicate significant difference among treatments (Tukey’s test, *p* ≤ 0.05).

Plants treated with film 9 developed roots significantly longer and wider than untreated plants. The same treatment tended to produce larger roots compared with the other formulations and with plants treated with both bacterial concentrations (Figure 4).

**Figure 5:**
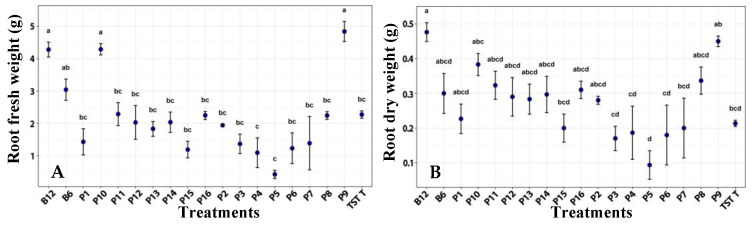
Root fresh weight (**A**) and dry weight (**B**) of tomato plants under a photoperiod, after the application of treatments. B12 (*B. subtilis* 1 × 10^12^ CFU/mL), B6 (*B. subtilis* 1 × 106 CFU/mL), TST T (untreated plants), P1–P16 (film formulation 1–16). Values are mean ± SE (n = 10). Different letters indicate significant difference among treatments (Tukey’s test, *p* ≤ 0.05).

Root fresh and dry weights were significantly higher in plants treated with film 9 than in untreated plants. Film 9 also tended to increase root fresh and dry weights compared with plants treated with other films (Figure 5).

### 2.2. Evaluation of Root Films for the B. subtilis Establishment on Tomato Roots

**Figure 6:**
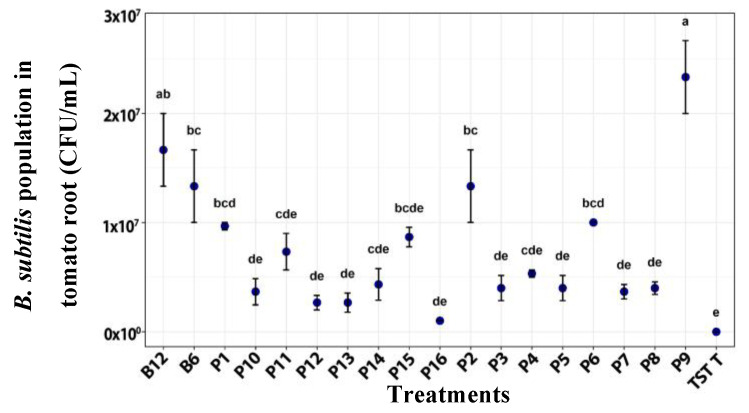
*B. subtilis* population on roots of tomato plants after the application of treatments. B12 (*B. subtilis* 1 × 10^12^ CFU/mL), B6 (*B. subtilis* 1 × 106 CFU/mL), TST T (untreated plants), P1–P16 (film formulation 1–16). Values are mean ± SE (n = 10). Different letters indicate significant difference among treatments (Tukey’s test, *p* ≤ 0.05).

Plants treated with film 9 showed the highest *B. subtilis* concentration on roots, followed by B12, compared with the other treatments; both share the same bacterial concentration used (1 × 10^12^ CFU/mL) (Figure 6).

### 2.3. Use of Film 9 as a Culture Medium for B. subtilis

**Figure 7:**
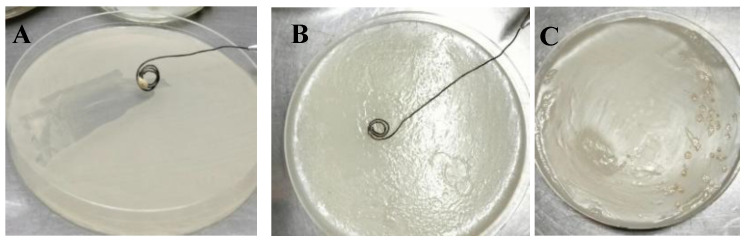
Growth of *B. subtilis*: (**A**) mass streak inoculation on PDA medium; (**B**) mass streak inoculation on medium containing the base film formulation; and (**C**) streak inoculation on medium containing base the film formulation.

*B. subtilis* grew in 100% of plates on both the film-based medium and PDA. Although growth was more abundant on PDA, it was evident on the base film 9 medium as well, as shown in panels B and C (Figure 7).

### 2.4. Film Degradation in Soil

**Figure 8:**
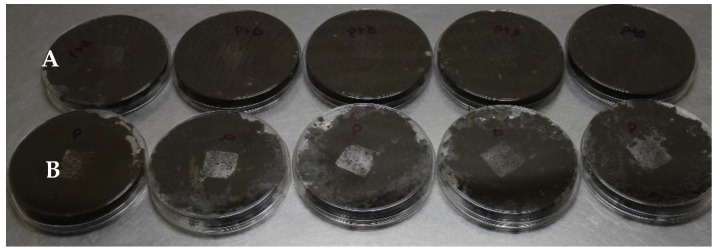
Film degradation in soil: (**A**) film without *B. subtilis*; (**B**) film with *B. subtilis*.

After 15 days of placing the film pieces in the soil, the piece the film 9 containing *B. subtilis* GOS 01 B-67748 showed evident degradation and was barely visible compared with the film without bacteria (Figure 8).

### 2.5. Viability of B. subtilis in the Film Formulation

**Figure 9:**
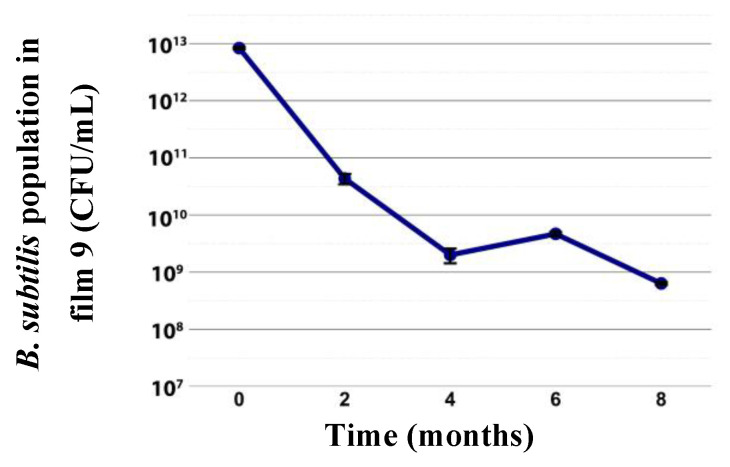
*B. subtilis* concentration (CFU/mL) in film formulation 9 during storage at 4 °C.

Over 8 months of storage of film 9, bacterial viability was maintained, though it decreased gradually. Initially at ~10^12^ CFU/mL, it decreased to ~10^10^ CFU/mL by month 2; at months 6 and 8 the decline was more gradual (~10^9^ to ~10^8^ CFU/mL) (Figure 9).

### 2.6. Molecular and Phylogenetic Analysis of B. subtilis Isolated from Tomato Root

**Figure 10:**
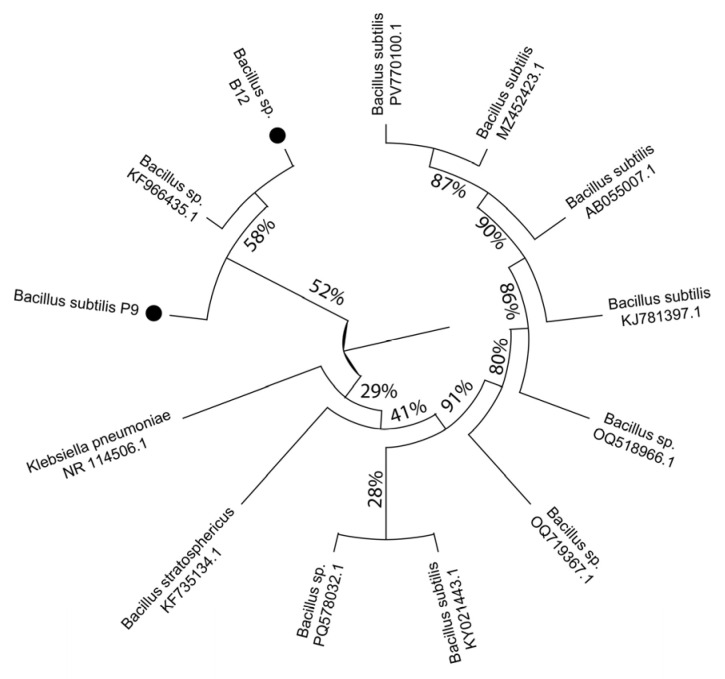
Phylogram of *B. subtilis* isolates. The phylogram was obtained by maximum-likelihood analysis of 16S sequences from 13 taxa. *Klebsiella pneumoniae* (Schroeter 1886) (NR 114506.1) was used as an outgroup. Numbers on branches are bootstrap values. Branch length is measured as substitutions per site. Bootstrap values ≥ 70% are shown. The sequence obtained in this study is indicated by a black circle.

Reference *Bacillus* spp. isolated from roots treated with film 9 and B12 taxa showed a significant evolutionary distance from the isolates, with the closest taxon being *Bacillus* sp. KF966435.1 (bootstrap ≥ 58%). Both isolates show a phylogenetic relationship with *B. subtilis*, although on independent branches with bootstrap values of 52% and 58%, respectively. While these values can be interpreted as moderate, they confirm a reliable proximity to the *B. subtilis* clade and suggest a distinct evolutionary trajectory relative to GenBank sequences consulted (Figure 10). 

## 3. Discussion

Edible-film formulations have incorporated microorganisms for diverse functionalities—maintaining viability, serving as carriers of phytopathogen control agents, among others. In this study, film formulation 9, composed of 0.6% guar gum, 0.15% candelilla wax, 0.15% glycerol, and 20% *B. subtilis* (10^12^ CFU/mL), was applied to tomato seedling root balls prior to transplanting. After 60 days, the film increased total dry weight (shoot + root) by 249% relative to the control. These results exceed those reported by [35] who used *B. subtilis* EA-CB0575 inoculated on tomato roots (10^8^ CFU/mL, 1-h root soak) in commercial soil under greenhouse conditions, achieving an 82.4% increase in total dry weight at 60 days after inoculation. The bacterium was predominantly localized in the upper and lower root zones, correlating with growth promotion.

Previous studies with *Bacillus amyloliquefaciens* (ex Fukomoto 1943) MBI 600 also showed positive effects in tomato. The bacterium was applied (10^10^ CFU/mL) by drench to pots (80 cm^3^) with peat:perlite (5:1), followed by a 10-day post-sowing application (10^7^ CFU/mL). This approach increased plant height (20.91%), root length (13.63%), and shoot fresh weight (115.32%), but reduced root fresh weight (−21%) compared with controls [36]. In contrast, with film 9 applied to the root ball and plants grown in loam soil, increases were greater for plant height (33.64%), root length (55%), shoot fresh weight (97.8%), and root fresh weight (113.36%) relative to controls. In tomato inoculated with *B. subtilis* NCD-2 at transplant (3 mL at 10^9^ CFU/mL) in pots with soil:vermiculite:peat (2:2:1 *v*/*v*/*v*) under a 16/8 h photoperiod, the bacterium promoted growth: at 35 days post-inoculation, plant height increased 19%, shoot fresh weight 27.25%, shoot dry weight 20.06%, root fresh weight 72.31%, and root dry weight 14.39% compared with controls; *B. subtilis* NCD-2 in rhizospheric soil reached 6.98 pg/g, evidencing establishment [17]. With film 9 applied once to the root ball and plants grown in loam, more pronounced effects on shoot and root biomass were observed at 60 days: plant height rose 33.64%; shoot fresh and dry weights increased 97.8% and 135.7%, respectively; root fresh and dry weights increased 113.3% and 114%, respectively, versus controls. de O Nunes et al., 2023 [28] evaluated *B. subtilis* FMCH002 (1 × 10^8^ CFU/mL), inoculating the growth substrate (2.5 mL), transplanting to rhizotrons (soil:commercial substrate 3:1 *v*/*v*) with additional bacterial applications (10 mL) at 10 and 20 days after transplant. After 32 days, FMCH002 increased plant height by 17.3%, shoot fresh weight 26.9%, shoot dry weight 36.4%, root dry weight 177.2%, and root length 37.1%, although root fresh weight decreased by 25%. In contrast, in the present work, a single root-ball application of film 9 with *B. subtilis* GOS 01 B-67748 increased height by 33.6%, shoot fresh weight by 97.8%, shoot dry weight by 135.7%, root fresh weight by 113.3%, root dry weight by 114%, and root length by 55%.

Regarding root bacterial load, *B. subtilis* MBI600 declined rapidly after inoculation across substrates. Initially at 2 × 10^10^ CFU/cm root, after 5 days it dropped to 2 × 10^5^ CFU under gnotobiotic conditions, 3.2 × 10^5^ CFU/cm in commercial peat, 4 × 10^4^ CFU/cm in garden soil, and 3 × 10^5^ CFU/cm in hydroponic cubes; by 20 days, concentrations declined markedly in each system (3 × 10^2^, 2.5 × 10^2^, 1.7 × 10^2^, and 4 × 10^2^ CFU/cm, respectively) [37]. With film 9 applied to tomato roots grown in loam soil, *B. subtilis* remained at 2.3 × 10^7^ CFU/g root at 60 days.

Similarly, under gnotobiotic conditions (20 cm^3^ Perloflor^®^ + 30 g pure sea sand + 10% *v/v* nutrient solution in a 220 × 25 mm glass tube), 2 mL of *B. amyloliquefaciens* MBI600 (2 × 10^10^ CFU/mL) were added at planting. At 15 days, roots reached 13 × 10^3^ CFU/mL [36]. In contrast, in this work, roots treated with film 9 carrying *B. subtilis* presented 2.3 × 10^7^ CFU/g at 60 days, evidencing higher concentration, persistence, and establishment.

Combinations of *B. subtilis* with other *Bacillus* species have also been evaluated. For example, *B. subtilis* + *B. licheniformis* [28] yielded root *Bacillus* concentrations up to 8.35 × 10^9^ CFU/g a few days after transplant, albeit with high variability among treatments (10^3^–10^7^ CFU/g). Here, although *B. subtilis* populations were lower (2.33 × 10^7^ CFU/g), they remained stable at 60 days, indicating that the film favored long-term persistence an advantage over short-lived conventional inoculations.

Encapsulation of *B. subtilis* CC-pg104 in sodium alginate crosslinked with CaCl_2_ (1.5%) prepared with glycerol (30%), humic acids (10%), and alginate (2%), followed by addition of a pellet from 250 mL of *B. subtilis* (2 × 10^10^ CFU/mL) to form beads and lyophilization, was applied (100 mg) to lettuce seedlings under gnotobiotic conditions. Treatments included free cells (1 mL, 1 × 10^8^ CFU) and empty capsules. Encapsulation increased lettuce root length (~76%) and shoot (~64%) relative to the no-bacteria control, while compared with free cells only shoot increased (~28%) after 21 days. Rhizosphere counts were 2 × 10^3^ CFU/cm for free cells and 4 × 10^7^ CFU/cm for encapsulated bacteria; product viability remained at 5.3 × 10^8^ CFU/mL for 5 months [30]. In tomato roots at 60 days, concentrations were similar; moreover, over a longer period (8 months) *B. subtilis* concentration in film 9 stored at 4 °C remained at 6.3 × 10^8^ CFU/mL. In another study, *B. subtilis* PTS-394 (~5 × 10^7^ CFU/mL; 20 mL) was added to substrate (paddy soil:vermiculite:organic fertilizer 1:2:1, w/w) of tomato with 4 true leaves. Thirty days after transplant, plant height increased 8.9% and root fresh weight 18.30% versus controls, and at 21 days root counts reached 2 × 10^6^ CFU/g [29]. With film 9, greater increases were achieved (height 33.6%; root fresh weight 113.3%) and rhizoplane counts reached 2.3 × 10^7^ CFU/g at 60 days.

Regarding shelf life of film formulations, a preformed cassava starch film (4%) with glycerol (1.5%) and CMC (2%) plus *B. amyloliquefaciens* Y11 or *B. velezensis* Y12 (3%) maintained bacteria within 10^6^–10^7^ CFU/g when analyzed every 5 days up to day 30 [38]. Although here bacterial concentration in film 9 was assessed in liquid, after a longer storage period (8 months) a high concentration (6.3 × 10^8^ CFU/mL) was preserved.

Some film formulations maintain viability of incorporated strains; for example, a 100% *Aloe vera* (L.) Burm.f., 1768 film with 1.0 g·L^−1^ glycerol and 0.1 M glucose increased viability of *L. paracasei* TEP6 (10.9 log CFU·g^−1^) [23]. On the base formulation of film 9, *B. subtilis* GOS 01 B-67748 showed limited growth; however, this suggests it may have used one or more ingredients as carbon and energy sources. *B. subtilis* P2-5 produces *β*-mannanase that hydrolyzes mannan present in guar gum [39]. Likewise, *B. subtilis* subsp. inaquosorum CSB31 produces an extremely alkaline mannanase (MnB31), NaCl-tolerant (10%), urea-stable (3 M), and protease-resistant [40]. These features reflect enzyme adaptation to common soil conditions, demonstrating bacterial metabolic tolerance. Similarly, *Streptomyces* sp. CS428 synthesizes *β*-mannanase that hydrolyzes carob galactomannan, releasing mannobiose, mannotriose, mannose, and various manno-oligosaccharides [41]. Such mono- and oligosaccharides can be utilized by microbial metabolism [42], indicating that *B. subtilis* likely used these compounds as energy sources in the guar-gum medium of film 9. Guar gum is also recognized as a gelling agent in media for fungal and bacterial growth, evidencing compatibility with beneficial microorganisms [43].

Regarding degradability, after 15 days film 9 was scarcely perceptible in soil compared with film without bacteria. A similar behavior was observed with cassava starch (4%), glycerol (1.5%), and CMC (2%) films containing *B. amyloliquefaciens* Y11 or *B. velezensis* Y12 (3%): 2 × 2 cm pieces became undetectable by day 15 in soil [38].

## 4. Materials and Methods

### 4.1. Biological Material and Raw Materials

The *B. subtilis* strain GOS 01 B-67748, registered with the Northern Regional Research Laboratory, was used for its ability to promote tomato growth and control fungi such as *F. oxysporum*, *R. stolonifer*, *R. solani*, among others. This strain is part of the microorganism collection of the Phytopathology Laboratory, Instituto Politécnico Nacional, CIIDIR Michoacán Unit, Mexico. Tomato seeds were the Rio Grande variety (KristenSeed^®^, Jalisco, Mexico). Guar gum (Diquítra^®^, Mumbai, India), glycerol (≥99.5% purity, J.T. Baker^®^, Phillipsburg, NJ, USA), and food-grade refined candelilla wax (Abreiko, Jalisco, Mexico) were used for the biofilm formulations.

### 4.2. Design and Preparation of Film Formulations

Film formulations were designed with guar gum, candelilla wax, glycerol, and *B. subtilis* using a 2^4^ factorial design in Design-Expert^®^ (version 12, Stat-Ease, Minneapolis, MN, USA), where the ingredients were the factors, each at two levels (high and low) (Table 1).

*Bacillus subtilis* suspensions were prepared by mass culturing on sterile Potato Dextrose Agar (PDA) plates and incubation at 37 °C (Thermo Scientific^®^, Langenselbold, Hesse, Germany) for 48 h. Biomass was collected with a sterile loop and suspended in sterile distilled water to 1 × 10^6^ or 1 × 10^12^ CFU/mL, verified by absorbance of 0.50 or 1.00 at 520 nm, previously calibrated with serial dilutions and optical density analyses on a UV-Vis spectrophotometer Lambda 2 (PerkinElmer^®^, Überlingen, Baden-Württemberg, Germany). Suspensions were prepared immediately before incorporation into the films.

Films were prepared by melting candelilla wax in distilled water at 80 °C in a 1-L blender glass jar; once melted, glycerol and guar gum were added, followed by high-speed homogenization for 3 min in a blender (Oster Classic^®^, Atlanta, GA, USA) to form an emulsion. This mixture was sterilized at 121 °C for 15 min and, after cooling to 30 °C, the bacterial suspensions were added.

### 4.3. Evaluation of Root Films for the Promotion of Tomato Growth

Tomato seeds were disinfected with 3% sodium hypochlorite for 5 min and rinsed four times with sterile distilled water. Seeds were sown in loam substrate, sterilized at 121 °C for 1 h over three consecutive days, in a 200-cell germination tray (54.5 cm × 28.8 cm × 3.5 cm; 23 mL per cell), and maintained under a photoperiod (6 h light/8 h dark). When seedlings developed 4 true leaves, their root balls were dipped for 5 s in the film formulations and then transplanted to sterile 1-L pots containing 500 g of sterile loam substrate as above. Ten seedlings per treatment were maintained under a 16 h light/8 h dark photoperiod. Treatments were the 16 formulations (P1–P16); controls were plants treated with *B. subtilis* suspensions (1 × 10^6^ and 1 × 10^12^ CFU/mL) and untreated plants (TST). After 60 days, plant height; root growth (length and width measured with a 5-m tape, Cadena^®^, MGA 5020, Taipei, Taiwan); number of well-developed leaves; and chlorophyll content (measured with a SPAD 502 meter, Konica Minolta, Marunouchi, Chiyoda-ku, Tokyo, Japan). Shoot and root fresh weights were determined using an analytical balance (Electronic Balance Kyoto, Japan). Shoot and root dry weights were determined using an analytical balance after drying at 105 °C for five days in a forced convection oven, (Terlab^®^, Zapopan, Jalisco, Mexico). A total of ten plants per treatment were considered.

### 4.4. Evaluation of Root Films for the B. subtilis Establishment on Tomato Roots

The establishment of *B. subtilis* on roots was also assessed 60 days after treatment application. For each treatment, 1 g of root was serially diluted in 20-mL tubes with 9 mL sterile distilled water. Then, 1 mL of sample was inoculated onto PDA, spread evenly, air-dried in a laminar-flow hood for 30 min, and incubated at 37 °C for 48 h. Plating for each treatment was performed in triplicate. Colonies with growth characteristics consistent with *B. subtilis* were selected following according to [44], smeared onto slides, and examined under a compound microscope at 100× magnification (Carl Zeiss^®^ Oberkochen, Bande-Württember, Germany) for morphological identification following the criteria described in [44].

### 4.5. Use of Film 9 as a Culture Medium for B. subtilis

The film formulation that was most effective at promoting growth and maintaining a higher *B. subtilis* concentration on tomato roots was selected for evaluation on plants placed at experimental and commercial greenhouse level, and as a culture medium for the bacterium.

Formulation 9 was prepared as a culture medium with the previously described ingredients and concentrations, plus 15 g/L bacteriological agar. Agar was dissolved by microwave heating for 3 min at power level 10 (≈100 °C), sterilized at 121 °C for 15 min, poured (20 mL) into sterile plastic Petri dishes (90 × 15 mm), and allowed to solidify. Then, 1 mL of *B. subtilis* suspension (1 × 10^12^ CFU/mL) was spread and air-dried in a laminar-flow hood for 30 min. Plates were incubated at 37 °C for 72 h. As a control, the bacterium was cultured on PDA. Five replicates per medium (P9 and PDA) were included, and bacterial growth was recorded.

### 4.6. Degradation Film 9 in Soil

Degradation of the preformed film in sterile soil was determined using formulation P9 due to its capacity to promote tomato plant growth. Twenty milliliters of formulation 9 were poured into sterile Petri dishes (90 × 15 mm) within a laminar-flow hood; covered plates were placed in a convection oven at 37 °C to form a uniform sheet. In the hood, 2-cm^2^ pieces were cut with a sterile scalpel and placed at the bottom of sterile Petri dishes. Twenty grams of sterile loam soil (121 °C, 1 h, on three consecutive days; pH 7.0) were added on top, and samples were maintained at 60% moisture and 25 ± 2 °C until degradation was observed. Treatments were soil with film 9 (P9) and soil with base film (P). Five replicates per treatment were included.

### 4.7. Viability of B. subtilis in the Film Formulation

The emulsion corresponding to film 9 was prepared and stored at 4 °C for eight months. Each month, the viability of *B. subtilis* in the formulation was determined by serial dilutions. For this proposer, 1 mL the emulsion was taken, serially diluted, and plate on PDA medium as previously described. Three replicates were considered for each evaluation.

### 4.8. Molecular Analysis of B. subtilis Isolated from Tomato Root

To identify *B. subtilis* on tomato roots, colonies with morphological characteristics per [44] were selected from the plates used to determine *B. subtilis* concentration. A single colony was transferred to PDA and incubated at 37 °C for 48 h; the resulting growth was used for molecular identification. Colonies were from roots treated with film 9 and 1 × 10^12^ CFU/mL.

DNA was extracted using the Quick-DNA™ Fungal/Bacterial Miniprep Kit (Zymo Research^®^, Irvine, CA, USA) following the manufacturer’s instructions. DNA presence was verified by electrophoresis on 1% (*w*/*v*) agarose gels. The presence of DNA was verified by electrophoresis on 1% (*w*/*v*) agarose gels prepared in 1× TAE buffer and run at 90 V for 30 min. DNA bands were visualized under UV light using a BIORAD Universal Hood II (USA) photodocumenter after staining with GelRed™ nucleic acid stain (Biotium, Fremont, CA, USA).

The 16S gene was amplified by PCR using universal primers 16S-F (AGAGTTTGATCCTGGCTCAG, 23.24 nmol) and 16S-R (ACGGCTACCTTGTTACGACTT, 25.14 nmol) (T4 OLIGO^®^, Irapuato Gto, Mexico). The 25-µL reaction mixture contained 10 µL PCR Master Mix (Thermo Scientific^®^), 10 µL DNA, 2 µL Forward, 2 µL Reverse, and 10 µL sterile ultrapure water.

PCR was performed in a thermocycler (BIO-RAD T100™ Thermal Cycler, Singapore) with the following conditions: an initial denaturation at 95 °C for 3 min, followed by 35 cycles of denaturation at 95 °C for 30 s, annealing at 55 °C for 30 s, and extension at 72 °C for 1 min, with a final extension at 72 °C for 10 min. To confirm amplicons, 2 µL of PCR product were run on 1% agarose gels at 90 V and 100 mA for 30 min and visualized in a photodocumenter (BIO-RAD^®^ Universal Hood II, Hercules, CA, USA).

### 4.9. Phylogenetic Analysis of B. subtilis Isolated from Tomato Root

After amplification was verified on 1.1% agarose gels, samples were sent to Macrogen^®^ (Seoul, Republic of Korea) for Sanger sequencing using an ABI 3730-xl DNA Analyzer (Applied Biosystems, Foster City, CA, USA; www.appliedbiosystems.com). Sequences of *B. subtilis* amplicons were identified by BLASTN against databases (GenBank, EMBL, DDBJ and UNITE for environmental sequences; http://unite.ut.ee/). All sequences were compared with GenBank using BLAST; the best match with maximum identity and E-value was recorded. A *B. subtilis* subspecies sequence was used as a reference group.

Sequences were processed to remove low-quality regions from forward and reverse reads and assembled into contigs using BioEdit (version 7.0.5.2). Assembled contigs were subjected to a BLAST search in NCBI (BLASST version 2.13.0) to corroborate identity and determine similarity with published sequences, selecting those with at least 98% identity. Amplicon sequences were compared with five similar sequences each, selected from public databases (NCBI) and represented by their GenBank IDs. Phylogenetic analysis of *B. subtilis* sequences was performed with MEGA 11 [45,46]. The consensus phylogram was constructed with the PHYLIP 3.6 package (consensus program) using the maximum-likelihood method on 16S sequences from 10 taxa. *Klebsiella pneumoniae* (NR 114506.1) was used as the outgroup. Numbers on branches correspond to bootstrap values. Branch lengths are measured as substitutions per site. Bootstrap values ≥ 70% are displayed. The sequences of the bacterial species obtained in this study are marked by a black circle (*Bacillus subtilis* BF 16S and *Bacillus* sp. CB 16S).

### 4.10. Statistical Analysis

Data were analyzed using ANOVA to detect significant differences. Mean separation was performed with Tukey’s test (*p* ≤ 0.05) using R (version 4.1.1) within RStudio for Windows 10.

## 5. Conclusions

The *B. subtilis* root film (formulation 9) composed of 0.6% guar gum, 0.15% candelilla wax, 0.15% glycerol, and 20% *B. subtilis* suspension at 1 × 10^12^ CFU/mL, produced the greatest effect on plant growth promotion, increasing shoot and root biomass in tomato. It also enabled higher *B. subtilis* colonization on roots than treatment with bacteria alone. These results indicate that the biofilm not only enhances bacterial colonization but also favors plant development, demonstrating its potential as a bioinoculant in tomato cultivation.

Within the formulation 9 matrix, *B. subtilis* remained at high concentration over a prolonged period (8 months) and grew when cultured on a medium prepared from the same film. Rapid degradation of the root film in soil was observed, suggesting that the bacterium can utilize some ingredients as carbon and energy sources. This capacity to degrade an innocuous material is a useful strategy to potentiate beneficial functions and represents a reliable agroecological alternative for large-scale application in tomato crops.

This section is not mandatory but can be added to the manuscript if the discussion is unusually long or complex.

## Figures and Tables

**Table 1:** Design of film formulations. Each formulation (P1–P16) contains the indicated percentages of guar gum, candelilla wax, and glycerol, plus *B. subtilis* suspension at 20% (*v*/*v*) with the stated concentration.

Film	Guar Gum (%, *w*/*v*)	Candelilla Wax (%, *w*/*v*)	Glycerol (%, *w*/*v*)	*B. subtilis* (20% *v*/*v*, CFU/mL)
1	0.30	0.30	0.30	1 × 10^12^
2	0.60	0.15	0.15	1 × 10^6^
3	0.30	0.15	0.30	1 × 10^12^
4	0.60	0.30	0.15	1 × 10^6^
5	0.60	0.15	0.30	1 × 10^6^
6	0.30	0.30	0.15	1 × 10^12^
7	0.30	0.15	0.15	1 × 10^12^
8	0.60	0.30	0.30	1 × 10^6^
9	0.60	0.15	0.15	1 × 10^12^
10	0.30	0.30	0.30	1 × 10^6^
11	0.60	0.30	0.15	1 × 10^12^
12	0.30	0.15	0.30	1 × 10^6^
13	0.60	0.15	0.30	1 × 10^12^
14	0.30	0.30	0.15	1 × 10^6^
15	0.60	0.30	0.30	1 × 10^12^
16	0.30	0.15	0.15	1 × 10^6^

## Data Availability

The original contributions presented in the study are included in the article, further inquiries can be directed to the corresponding authors.
